# Chronic dislocation of proximal interphalangeal joint with mallet finger: A case report

**DOI:** 10.1186/1757-1626-1-201

**Published:** 2008-10-02

**Authors:** Paisal Hussin, Subramaniam Mahendran, Eng Seng Ng

**Affiliations:** 1 Orthopaedic Department, Faculty of Medicine and Health Sciences, Universiti Putra Malaysia, Serdang, Selangor, Malaysia; 2 Orthopaedic Department, Faculty of Medicine, Universiti Malaya, 50603 Kuala Lumpur, Malaysia

## Abstract

We report a case of chronic dislocation of proximal interphalangeal joint with mallet finger.

## Introduction

Simultaneous occurrence of proximal interphalangeal joint dislocation and mallet deformity on the same finger are rare. They are commonly seen as a result of sports injuries. Patients usually present early for treatment due to gross deformity and pain of affected finger. We would like to report a variant of proximal interphalangeal joint dislocation associated with bony mallet injury.

## Case report

A twenty five years old Indian male presented to our clinic with painful swelling over his right ring finger. He was involved in a motor vehicle accident three weeks prior to the visit and sustained injury to his right ring finger. He didn't seek any medical treatment initially as he had minimal pain and was able to function with minimal difficulties then. However as the pain and swelling had been persistent, he had decided to come for medical attention.

On examination, the right ring finger was swollen with tenderness elicited mainly around the proximal and distal interphalangeal joints.

There was a mallet deformity and he was not able to actively extend the affected joint.

The proximal interphalangeal joint was grossly swollen and deformed with no active or passive range of movement.

**Figure 1:**
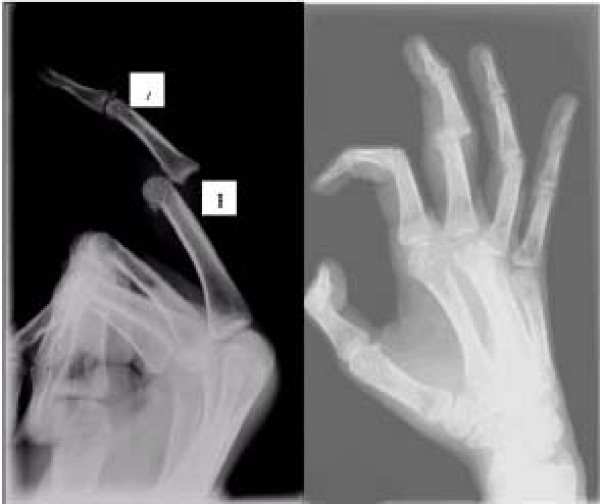
**X-ray of lateral and oblique view. Note the avulsed bony insertion of extensor of the distal phalanx.** (A). volar plate fracture (B).

X ray showed dorsal dislocation of proximal interphalangeal joint with avulsed bony fragment from distal phalanx of the right ring finger. (Figure 1)

Initially a closed reduction was attempted under digital block but was unsuccesful.

We then proceeded with an open reduction under general anaesthesia.

**Figure 2:**
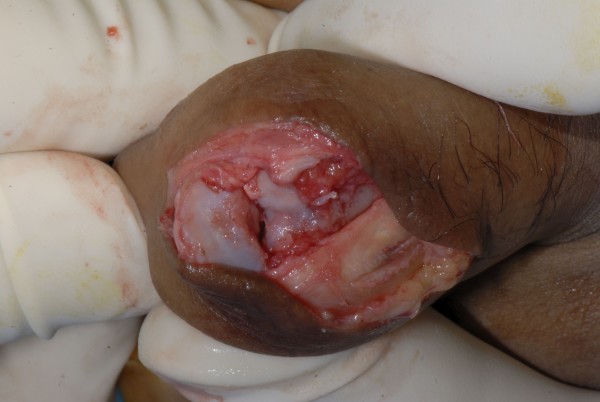
Showing dorsal dislocation of the joint. The joint was filled with fibrous tissue.

Intra-operatively, we noted a volar plate fracture with dorsal dislocation of the proximal interphalangeal joint and marked fibrosis. (Figure 2).

**Figure 3:**
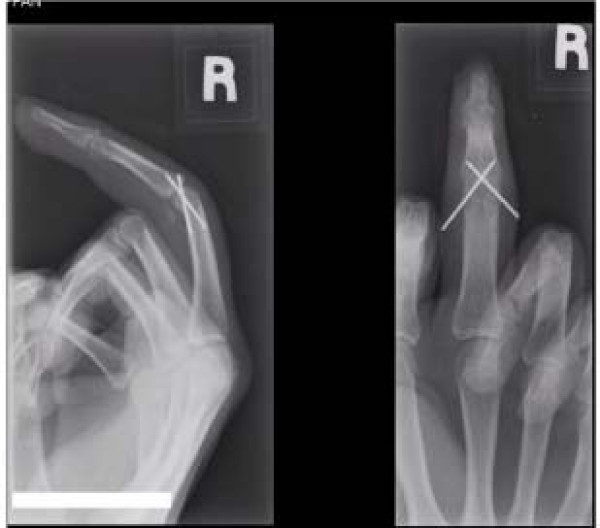
Post operative x-rays showing the reduced PIP joint and stabilized with K wires.

Fibrotic tissues were removed. Dislocation was reduced and stabilized using wire. (Figure 3).

The Mallet injury was treated with mallet splint.

K-wire was removed after 3 weeks and aggressive mobilization of PIP was started. The Mallet splint was kept for 6 weeks.

On latest review 3 months after the operation, there was no extension lag of DIP joint with flexion (0–40°)

The PIP joint was stable but stiff with flexion of 10°–90°. He was advice on continuous exercise.

## Discussion

It is rare to have mallet fracture and dislocation of proximal interphalangeal joint occurring simultaneously on the same finger. These injuries are caused by hyperextension force on the finger. However in this patient the mallet fracture could have been resulted by sudden flexion during the extension of the distal phalanx.

In the normal practice it is quite rare to get chronic dislocation of proximal phalanx. Dislocation of proximal interphalangeal joint usually will be detected early as it will give distinct clinical picture. However, in this case the dislocation was picked later as the patient presented late for treatment.

Stability of the proximal interphalangeal joint is maintained by volar plate, collateral and extensor expansion. Dorsal dislocation is the commonest form. It is associated with volar plate or collateral ligament ruptures. Central slip of the extensor tendon can be rupture as well. In a more complex and irreducible type, the head of the proximal phalanx may be displaced between the volar plate and the flexor tendon which act like a buttonhole by tightly grasping the portion of the phalanx immediately behind the head, preventing closed reduction. Additionally, the distal attachment of the volar plate may rupture so that the volar plate becomes interposed between the joint surfaces. These dorsal dislocations require open reduction and repair.

Proximal interphalangeal joint can be either treated conservatively or operatively. Nonoperative devices such as dorsal blocking splint and extensor block pinning had been used previously. A number of operative interventions had been employed such as open reduction internal fixation, volar plate arthroplasty and superficialis tenodesis but the results are still questionable.

Stiffness of the joint is the most common complication of this injury. In this case, although the patient came late for treatment, result of treatment is good.

## Competing interests

The authors declare that they have no competing interests.

## Authors' contributions

PH and MS were recorded the patient data, took the photos and major contributor in writing the manuscript. NES was performed the intervention and revising the manuscript. All authors read and approved the final manuscript.

## Consent

Written informed consent was obtained from the patient for publication of this case report and accompanying images. A copy of the written consent is available for review by the Editor-in-Chief of this journal.
